# Modelling of icodextrin hydrolysis and kinetics during peritoneal dialysis

**DOI:** 10.1038/s41598-023-33480-w

**Published:** 2023-04-21

**Authors:** Joanna Stachowska-Pietka, Jacek Waniewski, Anna Olszowska, Elvia Garcia-Lopez, Zofia Wankowicz, Bengt Lindholm

**Affiliations:** 1 Nalecz Institute of Biocybernetics and Biomedical Engineering, Polish Academy of Sciences, Ks. Trojdena 4, 02-109 Warsaw, Poland; 2 Military Institute of Medicine, Central Hospital of the Ministry of Public Defence, Warsaw, Poland; 3 Department of Clinical Science, Intervention and Technology, Division of Renal Medicine and Baxter Novum, Karolinska Institutet, Stockholm, Sweden

**Keywords:** Renal replacement therapy, Biomedical engineering

## Abstract

In peritoneal dialysis, ultrafiltration is achieved by adding an osmotic agent into the dialysis fluid. During an exchange with icodextrin-based solution, polysaccharide chains are degraded by α-amylase activity in dialysate, influencing its osmotic properties. We modelled water and solute removal taking into account degradation by α-amylase and absorption of icodextrin from the peritoneal cavity. Data from 16 h dwells with icodextrin-based solution in 11 patients (3 icodextrin-exposed, 8 icodextrin-naïve at the start of the study) on dialysate volume, dialysate concentrations of glucose, urea, creatinine and α-amylase, and dialysate and blood concentrations of seven molecular weight fractions of icodextrin were analysed. The three-pore model was extended to describe hydrolysis of icodextrin by α-amylase. The extended model accurately predicted kinetics of ultrafiltration, small solutes and icodextrin fractions in dialysate, indicating differences in degradation kinetics between icodextrin-naïve and icodextrin-exposed patients. In addition, the model provided information on the patterns of icodextrin degradation caused by α-amylase. Modelling of icodextrin kinetics using an extended three-pore model that takes into account absorption of icodextrin and changes in α-amylase activity in the dialysate provided accurate description of peritoneal transport and information on patterns of icodextrin hydrolysis during long icodextrin dwells.

## Introduction

In peritoneal dialysis (PD), the removal of water by transcapillary ultrafiltration depends on the osmotic force exerted across the peritoneal barrier by the osmotic agent(s) in the dialysis fluid. In conventional dialysis solutions, glucose (at different concentrations) is used as an osmotic agent. However, glucose diffuses from dialysate into the peritoneal tissue and further to the circulation, resulting in the dissipation of the osmotic gradient during the peritoneal dwell, which shortens the effective ultrafiltration period, especially in patients with fast solute transfer rate. Icodextrin is an alternative and much larger osmotic agent that due to its ability to provide sustained ultrafiltration, is used for the long exchanges, which is an important feature, especially in patients with fast solute transfer rate. Icodextrin is comprised of a mixture of different glucose polymers (dextrin molecules, ranging in size from 2 to about 1000 D-glucose units) derived from hydrolysed corn starch. The reported weight-average molecular weight, MW, of icodextrin ranges between 12–20 kDa, and the number-average molecular weight is about 5–6.5 kDa with polydispersity index close to 2^1,2^.

The D-glucose units of the polymer chains in the icodextrin-based solution are mainly (over 90%) linked by α – 1,4 glycosidic bonds that are degraded by α-amylase. This enzyme takes part in the process of starch digestion. Besides human saliva, it is present also in plasma and peritoneal tissue and slowly appears in dialysate during the peritoneal dialysis dwell. In the presence of α-amylase, icodextrin is degraded to shorter oligosaccharide chains. Clinical and experimental in vitro and ex vivo studies showed that icodextrin ultimately is hydrolysed mainly to maltose (G2), maltotriose (G3) and maltotetraose (G4)^3–5^. The hydrolysis of icodextrin caused by α-amylase activity changes the composition of dialysate, leading to greater content of shorter polymers, thereby altering the transport properties and effective osmolality of the icodextrin-based dialysis solution.

Various mathematical approaches have been proposed to model peritoneal transport. Their ability to describe and predict fluid and solute transport has been validated based on clinical and experimental data for glucose-based dialysis solutions^6–11^. In contrast, there are few studies published so far attempting to model fluid and solute transport during peritoneal dwells with icodextrin used as an osmotic agent. The first attempt in this field was done by Rippe and Levin^12^. They applied the original three-pore model to show the impact on water removal during dwells with icodextrin of various factors such as differences in instilled volume, fluid composition (different icodextrin concentration with or without glucose added; number of fractions considered in modelling of ultrafiltration), and peritoneal surface area^12^. In their theoretical study, icodextrin was modelled as a mixture of 8 reference molecules, so called fractions, and the clearances of these fractions were assumed to be inflated by 1.2 mL/min to account for their clearance to the peritoneal tissue. In a later work by Moberly et al. on the pharmacokinetics of icodextrin, although icodextrin fractions were measured, only total icodextrin absorption was modelled^13^. Besides these mostly theoretical approaches, several studies attempted to validate simulated results with data from icodextrin dwells, mainly regarding water removal^2,14–17^, and also urea and sodium transport^16,17^. However, so far only one study, by Akonur et al.^14^, analysed the impact of amylase activity on ultrafiltration and icodextrin absorption. Due to the lack of detailed animal or human data, the authors based their model on in vitro data^14^, and later applied it to simulate total carbohydrate absorption and total water removed in PD patients^15^.

Despite the wide usage of icodextrin solution, there is no study showing detailed fluid and solute kinetics–including transport of carbohydrates-during long dwells with icodextrin solution and verifying to which extent it can be predicted by the mathematical modelling. Clinical studies providing comprehensive data on intraperitoneal changes of all relevant factors, i.e., dialysate volume, concentrations of glucose poly-/oligomers and other solutes, and α-amylase activity, are scarce and therefore the full validation of mathematical models for icodextrin-based dialysis solutions has not been performed previously.

In the present study, the three-pore model of peritoneal transport (TPM) was extended (ETPM) by the description of icodextrin degradation by α-amylase in dialysate. In our study, the formulated and validated model of icodextrin hydrolysis by α-amylase in the peritoneal cavity was adjusted to the available data on the concentration of icodextrin fractions and their changes during peritoneal dwell. The extended model included identification of mechanisms involved in the osmotic activity of icodextrin and the impact of various patterns of amylase activity and their impact on the composition of dialysate during 16-h dwell with icodextrin-based solution^18,19^.

## Materials and methods

### Clinical data

Data from 11 patients undergoing PD with icodextrin solution were analysed. Three patients had used one exchange with icodextrin solution per day for 14.3 ± 5.1 months before the study (Ico+ group), whereas in 8 patients (icodextrin − naïve patients), it was their first usage of icodextrin solution (Ico− group).

The study complies with the Declaration of Helsinki principles and the Ethics Committee of Military Institute of Medicine, Warsaw, Poland approved the study protocol. Written informed consent was obtained from each patient after an explanation of the purpose of the study. A detailed description of the study protocol, clinical data, and data on the peritoneal transport of water, amylase and sodium can be found in^18,19^.

Briefly, each patient underwent a 16-h PD exchange with 2 L of 7.5% icodextrin solution (Extraneal®, Baxter Healthcare, Castlebar, Ireland) in which radiolabelled albumin (RISA) was added as a volume marker (125I-HSA, Serlab-125; Cis Biointernational, Gif-sur-Yvette, France). Dialysate samples (10 ml) were taken at 0, 3, 15, 30, 60, 90, 120, 180, 240, 480, 720, and 960 min of the exchange and blood samples (5 ml) were collected at 0, 15, 60, 120, 240, 480, 720, and 960 min of the exchange. To provide data for the calculation of residual volume, peritoneal cavity was rinsed with 2 L of fresh 1.36% glucose solution for 5 min after drain was finished. The samples were analysed for concentration of urea, creatinine, glucose, total icodextrin and icodextrin fractions.

The dialysate concentration of icodextrin polysaccharide chains was measured for low (LMW, oligosaccharides G2–G7, up to MW = 1153 Da) and high (HMW, polysaccharides higher than maltoheptatose G7) molecular weight polymers. Plasma concentration could only be established for part of LMW oligosaccharides (mainly G2 and G3, since concentrations of G4–G7 oligomers were too low to be measured in most samples) and not for HMW dextrins, using gel filtration high-performance liquid chromatography^3,18^. The α-amylase activity in blood and dialysate samples, further referred to as α-amylase concentration, were determined by a fully automated routine method from Konelab 20XT routine biochemical analyzer using p-nitrophenol maltoheptaoside as a substrate, as described previously in^5,20^.

### Calculations

Intraperitoneal volumes were estimated for each patient from the dilution of RISA with corrections applied for the absorption of RISA from the peritoneal cavity and sample volumes^21^. Concentrations of urea, creatinine, and glucose were expressed per plasma volume as described previously^22^. In contrast to glucose-based solutions, there is no evidence that icodextrin interferes with measurements of creatinine, and therefore no such correction was performed^23^.

**Figure 1:**
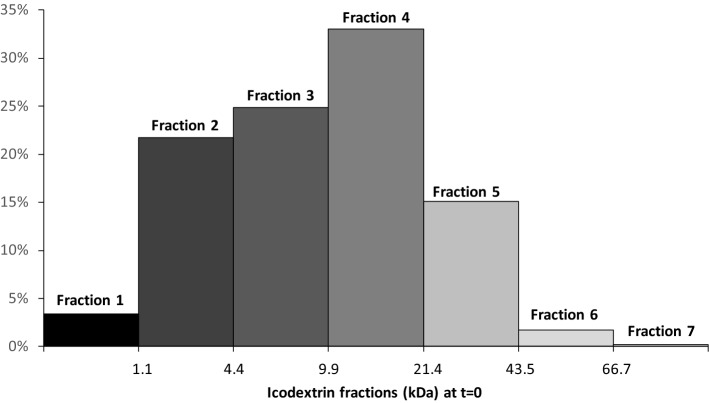
The relative (in percent) distribution of seven different molecular weight fractions in fresh icodextrin dialysis solution.

In dialysate, molecular weight distribution of icodextrin HMW and LMW polysaccharides was determined as described previously^3^. Briefly, at each sampling point, the concentration of icodextrin’s polysaccharides was calculated from chromatograms and aggregated into seven fractions (glucose polymer size classes) with the following MW cut-off values: up to 1.08 kDa (Fraction 1), 4.44 kDa (Fraction 2), 9.89 kDa (Fraction 3), 21.4 kDa (Fraction 4), 43.5 kDa (Fraction 5), 66.7 kDa (Fraction 6), and over 66.7 kDa (Fraction 7) as described in ^3^. This allows us to determine at each sampling point concentrations (in mg/mL) and relative (percentage) mass distributions of icodextrin fractions as presented in Fig. 1 for fresh dialysis solution.

The concentration of LMW icodextrin oligosaccharides in plasma could be determined for G2 and G3 but not for G4–G7 (non-detectable values). Therefore, for the purpose of the study, the concentration of LMWs in plasma was calculated as the sum of the concentration of G2–G7 oligomers, assuming concentrations of G4 and G5–G7 being 30% of that of G2 and G3 respectively, as reported for Ico+ and Ico− group by Garcia-Lopez et al.^3^. Moreover, since there is no direct evidence for the presence in plasma of polymers with higher MW or their concentration is very low (for G8–G10 up to 2–5% of total icodextrin present in plasma as reported in^24^), we assume that the plasma concentrations of HMW icodextrin polymers in plasma was for each fraction 2–5 equal to 0.08 mmol/L in Ico+ group, and 0 for fractions 2–5 in Ico− group and in fractions 6 and 7 in both groups. Thus we estimate that total icodextrin HMW polysaccharides concentration in plasma was on average 0.32 mmol/L, a value similar to that assumed by Vonesh (0.37 mmol/L) and Rippe et al. (0.35 mmol/L)^2,12^. Moreover, it has been shown previously that plasma concentration of LMW icodextrin polymers (dextrins from Fraction 1) increases during peritoneal dwell in both Ico+ and Ico− groups^18^. Therefore, the individual, time dependent profiles for the first fraction concentration in plasma were taken into account for the application of our model.

### The extended three-pore model of peritoneal transport

The original three-pore model (TPM) for peritoneal transport was applied according to the theory of the transport through the semipermeable membrane as proposed by Rippe et al.^25^ and later adapted to the peritoneal dwells with icodextrin-based solutions^2,12,16,17^. According to the TPM, fluid flow was considered as dependent on the difference between hydrostatic pressure and osmotic (crystalloid and colloid) pressure exerted by small solutes, icodextrin polymers, and proteins. Diffusive and convective solute transport across peritoneal membrane was considered for small solutes and icodextrin fractions. The absorption of fluid and solutes from the peritoneal cavity was also included in the model. Moreover, our data suggested that to accurately describe the kinetics of icodextrin fractions during peritoneal dialysis, TPM has to be extended by including a description of the degradation of icodextrin polymers due to the α-amylase activity in dialysate, as described in “Results” section. Modelling the dynamics of α-amylase activity is out of the scope of the present study, but we use the data on measured amylase activity to model icodextrin kinetics and to derive information on icodextrin hydrolysis during the peritoneal dwell (see Results).

A detailed description of TPM applied in this study is presented in the Supplementary material together with the Supplemental Figure 1 presenting schematic concept of the extended three-pore model (ETPM), whereas in Results detailed description of the extension of TPM is given.

### Numerical simulations, data fitting and statistics

The ETPM was solved using commercial software package MATLAB with the built-in function ‘ode45’, which is based on an explicit Runge–Kutta formula with variable step size. In order to fit a model to the data the following parameters were estimated: $L_{P} S$—hydraulic conductivity (peritoneal ultrafiltration coefficient), $\alpha_{SP}$ ($\alpha_{LP} = 1 - \alpha_{SP} - \alpha_{UP}$ was calculated and $\alpha_{UP}$ was kept equal to 0.02 as in^12,17^, where $\alpha_{UP} ,\alpha_{SP} ,\alpha_{LP}$ denotes fractional $L_{P} S$ attributed to ultrasmall, small and large pores, respectively), $PS$ (permeability-surface area product) for glucose, urea, creatinine, and icodextrin fractions. In addition, parameters related to the degradation kinetics were estimated: appearance $r_{i}^{j}$, for $i \le j$, and hydrolysis $h_{i}$ rate constants. The parameters $p_{i}$ describing hydrolysis probability/frequency for polymers belonging to Fraction *i* versus all icodextrin polymers were calculated based the estimated values of hydrolysis rate constants estimated for the ETPM. The time dependent hydrolytic clearances $H_{i}$ of icodextrin fractions were also evaluated.

The estimation of parameters was done using ‘lsqnonlin’ function implemented with the trust-region-reflective algorithm implemented to minimize sum of squared differences between modelled and measured values for intraperitoneal volume and concentration of glucose, urea, creatinine, and icodextrin fractions in dialysate. The concentration of solutes measured in plasma provided time-dependent (interpolated linearly between sampling points) profiles separately for each patient. The goodness of fit was expressed as an average relative error per measurement point.

The results were expressed as mean ± standard deviation (SD). Due to the low number of patients in Ico+ group we did not provide statistical comparison between Ico− and Ico+ . However, the obtained results were consistent for different parameters and remained in agreement with previous observations, as presented in discussion. The t-test or Wilcoxon rank test were used for the comparison between parameters of two models whenever appropriate. The ANOVA repeated measurements and post-hoc Tukey’s test were used to compare changes in time of hydrolytic clearances, $H_{i}$, and degradation frequencies, $p_{i}$. Statistical significance was accepted if *p* < 0.05.

## Results

A 16-h PD exchange with 2 L of 7.5% icodextrin solution was performed in 11 patients with (mean ± SD) age of 50.4 ± 18.3 years (median 59 years), body weight 73 ± 13 kg, height 164 ± 9 cm, and residual diuresis of 1.11 ± 1.16 L/day (median 0.7 L/day). Their mean time on PD was 26.9 ± 22.4 months (median 17 months). There were 4 patients with fast transfer (H), 4 with fast-average (HA), and 3 with slow-average (LA) of small solutes, according to peritoneal equilibration test performed 3–4 weeks before the study as described in^26^. Three patients had used icodextrin before (Ico+ group) and 8 patients were first time users of icodextrin (Ico− group). Clinical data on changes of intraperitoneal volume and concentration of icodextrin fractions used in the present study were published in part previously in^18,19^, whereas data on small solutes transport were not.

### Amylase kinetics in plasma and dialysate

**Figure 2:**
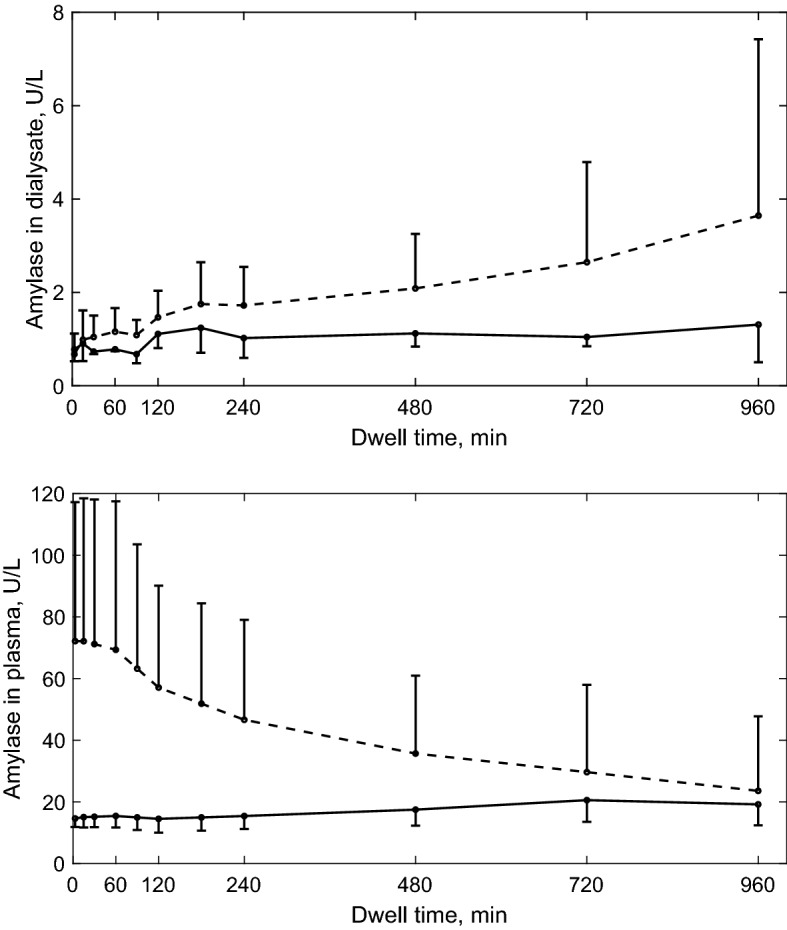
Mean ± SD values of α-amylase concentration (total activity) in dialysate (upper panel) and plasma (bottom panel) in Ico+ (solid line) and Ico− (dashed line) groups as a function of dwell time.

The α-amylase concentration in plasma tended to be initially higher in Ico− group than in Ico+ group, with decreasing trend and large variability during whole dwell period, probably related to the adaptation to the exchanges with icodextrin solution and its diffusion from plasma to the peritoneal tissue, Fig. 2, see also^18^. Interestingly, the α-amylase concentration in plasma after 16-h dwell in both groups converged to similar values, Fig. 2, bottom panel. The α-amylase concentration in dialysate over whole dwell period was, on average, increasing in the whole group, at different rates in Ico− and Ico+ groups (Fig. 2), see also^18^. The process of icodextrin hydrolysis is directly related to α-amylase level. Any changes in α-amylase concentration in the peritoneal cavity might influence icodextrin degradation in dialysate, and therefore the effect of hydrolysis has to be taken into account in the model.

### Theoretical model of icodextrin hydrolysis

In the present study, we used a deterministic approach to model the impact of amylase activity on the icodextrin concentration. To balance the complexity of the model with the accuracy of description we decided to use pseudo-first order kinetics. The proposed *extended three-pore model* (ETPM) provided a description of the icodextrin degradation kinetics caused by α-amylase activity adjusted to the measured fractions of icodextrin. The extension of TPM by including the effect of α-amylase on the concentration of dextrins was necessary to describe changes in icodextrin fractions during the dwell that were not related to the absorption of dextrins from the peritoneal cavity. Moreover, the dependence of degradation on the amylase content in dialysate has to be taken into account^14^, to describe the initial decrease followed by the later increase of the concentration of icodextrin Fraction 1, particularly pronounced in Ico− group. Furthermore, the assumption that plasma concentration of solutes remains stable during peritoneal dwell, that is typically postulated to model up to six-hours exchange using TPM, is not valid in case of long PD dwells. Therefore, the changes of concentration of small solutes in blood, including concentration of icodextrin Fraction 1 after 4 h of dwell due to appearance of small dextrins in dialysate (especially pronounced in Ico− group) and fluctuation of glucose level, were taken additionally into account in the model based on individual data. The mentioned changes were necessary for the proper description of the changes of the driving forces (osmotic pressure) to model peritoneal transport and degradation of icodextrin.

During hydrolysis, α-amylase breaks the long chains of icodextrin polysaccharides into shorter chains. One of the ways to model the changes of dextrin concentrations caused by hydrolysis is to use first order degradation kinetics assuming that the decrease of solute concentration over time caused by α-amylase activity occurs with degradation rate that is proportional to its concentration, in accordance with some degradation rate constant. So far various action patterns have been proposed to model hydrolysis of starch polymers by amylase, differing by the way they describe the probability of getting polymer with *i*-number of D-glucose units from the polymer having *j*-number of D-glucose units^27^. However, independently of the approach, and in agreement with experimental data, all the above-mentioned models assumed a non-zero probability of getting a new polymer from the same or “lower” fraction than the hydrolysed one. Therefore, the change of a particular icodextrin fraction, due to α-amylase activity, may not only decrease (caused by hydrolysis of polymers that are within this fraction) but also increase due to appearance of the hydrolysed polymers that were initially placed in another fraction or/and within the same fraction.

The overall change of the mass of icodextrin Fraction *i* for *i* = 1, …, 7 can be calculated according to the *extended model* as the result of peritoneal transport described by TPM (as typically modelled for glucose-based solutions) extended on the impact of icodextrin hydrolysis. Due to the presence of hydrolysis mass of Fraction *i* has to be increased for the inflow of new polymers from the upper fractions as well as the change caused by the hydrolysis of the polymers from the present fraction that might result in the increase or decrease of the solute mass. This can be formulated mathematically as follows:

1 $$\frac{{d\left( {V_{D} C_{{D,Ico_{i} }} } \right)}}{dt} = J_{{Ico_{i} ,SP}} + J_{{Ico_{i} ,LP}} - LC_{{D,Ico_{i} }} + \sum\limits_{j = i,...,7} {R_{i}^{j} C_{{D,Ico_{j} }} }$$

where $V_{D}$ is the intraperitoneal volume, $C_{{D,Ico_{i} }}$ denotes icodextrin *ith* fraction concentration, $L$ is peritoneal absorption rate, and $J_{{Ico_{i} ,SP}} ,J_{{Ico_{i} ,LP}}$ corresponds to the icodextrin’s *ith* fraction flow (mmol/min) through the small and large pores of the peritoneal membrane, respectively. The last term in Eq. (1) corresponds to the change of mass caused by the α-amylase induced hydrolysis, which leads to degradation of a polymer into two new polymers. Therefore, the change in mass of Fraction *i* related to the amylase activity depends on the appearance of new polymers due to the hydrolysis of the polymer from Fraction *j* (with rates constant $R_{i}^{j}$, $j > i$) , and also on the appearance related to hydrolysis within Fraction *i* (with rate constant $R_{i}^{i}$). The description of the transport processes according to the original three-pore model can be found in the Supplemental Materials.

In addition, both in-vitro and ex-vivo studies suggested that the degradation rate of icodextrin polymers is strongly related to the level of α-amylase concentration that is changing during the dwell. To account for this dependence the rate constant was assumed to be proportional to the amylase concentration in dialysate^28^. This leads to the model with pseudo-first kinetics given by Eq. (1) with time-dependent rates $R_{i}^{j} \left( t \right) = r_{i}^{j} \cdot V_{D} \left( t \right) \cdot C_{D,amylase} \left( t \right)$, where $r_{i}^{j}$ (in (1/min)/(U/mL) is the appearance rate constant related to the appearance of a polymer in Fraction *i* due the hydrolysis of the polymer from Fraction *j*. Therefore, in addition to the transport parameters required by TPM, the extended model requires additionally specification of the appearance rates constant $r_{i}^{j}$ for $j \ge i$.

### Theoretical considerations on icodextrin hydrolysis

**Figure 3:**
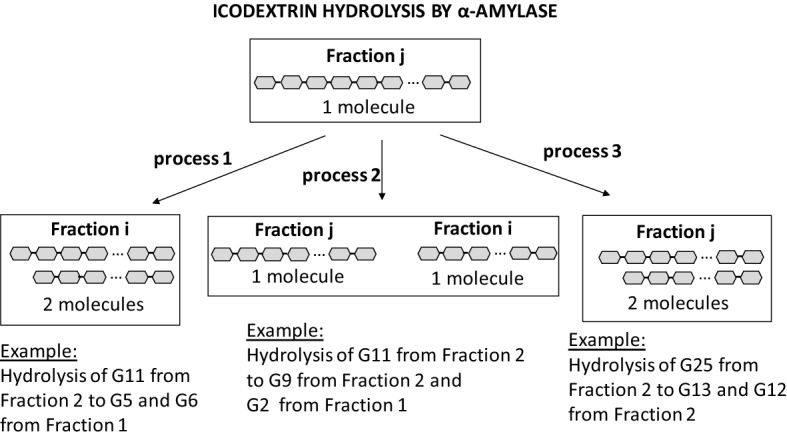
Three possible outcomes of the hydrolysis of icodextrin polymers and their impact on the change of molar mass of icodextrin fractions.

Let us consider hydrolysis of polymer belonging to the Fraction $i \ge 2$. The following three processes can be observed, as schematically presented in Fig. 3:

A polymer from Fraction *i* can be degraded either to:

A) Two polymers both belonging to the “lower” Fractions than *i* (process 1), or
B) Two polymers from which only one belongs to Fraction *i* (process 2), or
C) Two polymers both belonging to Fraction *i* (process 3).

In case of polymer belonging to Fraction 1 only third type of hydrolysis is possible, i.e., generation of two polymers both belonging to Fraction 1. Distinction between mentioned processes is important, since the molar mass of Fraction *i* may decrease only as a result of process 1, because process 2 does not lead to the decrease of molar mass within Fraction *i*. Moreover, the increase of molar mass of Fraction *i* depends on the concentration of polymers from fractions higher than *i* appearing as a result of process 1 (with appearance rate $r_{1,j}^{j}$) as well as from process 2 (with appearance rate $r_{2,i}^{j}$), and finally from the hydrolysis within the same fraction, i.e., from process 3 (with appearance rate $r_{3,i}^{i}$). In addition, since degradation of each icodextrin polymer leads to creation of two shorter polymers, one can calculate the mass of all hydrolysed polymers that initially were within Fraction *i* summarizing corresponding appearance rates of polymers to Fractions *j* = 1, …, *i*. Therefore, the hydrolysis rate constant at which polymers from Fraction *i* are degraded by amylase activity can be calculated from the appearance rates as $h_{i} = \sum\limits_{j = 1,...,i - 1} {\left( {0.5r_{1,j}^{i} + r_{2,j}^{i} } \right)} + r_{3,i}^{i}$ and the corresponding hydrolytic clearance of Fraction *i* is equal to $H_{i}^{{}} \left( t \right) = h_{i}^{{}} \cdot V_{D} \left( t \right) \cdot C_{D,amylase} \left( t \right)$.

Note, that in ETPM*,* described by Eq. (1), for $i < j$ the rate constant $r_{i}^{j}$ denotes appearance rate at which mass of icodextrin Fraction *i* increases as a result of hydrolysis of polymers from Fraction *j* to at least one polymer belonging into Fraction *i* (processes 1 and 2), and therefore $r_{i}^{j} = r_{1,i}^{j} + r_{2,i}^{j}$. In case of hydrolysis within the same fraction, the corresponding mass increases as a result of process 3 and decreases due to process 1 (for *i* > 1) leading to $r_{i}^{i} = \left( {r_{3,i}^{i} - 0.5\sum\limits_{j = 1,...,i - 1} {r_{1,j}^{i} } } \right)$ for *i* > 1. Applying the definition of hydrolysis rate, $h_{i}$, one can get that $r_{i}^{i} = \left( {h_{i} - \sum\limits_{j = 1,...,i - 1} {r_{j}^{i} } } \right)$ for $i > 1$.

Note that the mass of icodextrin Fraction *1* increases and new polymers appear in Fraction *1* not only due to the hydrolysis of polymers from Fractions 2–7 but also due to hydrolysis of the polymers already present in Fraction *1*. Moreover, since processes 1 and 2 are not present in this fraction, the decrease of icodextrin mass corresponding to Fraction 1 occurs only due to the absorption from the peritoneal cavity. Therefore, for icodextrin Fraction *1*: $r_{1}^{1} = r_{3,1}^{1}$.

### Numerical results

**Figure 4:**
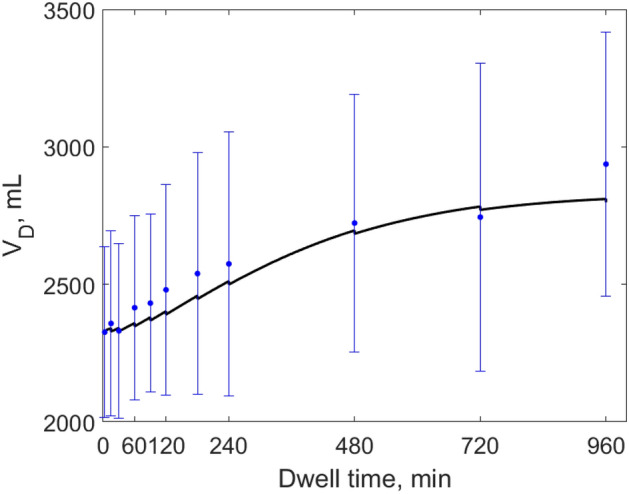
Measured values of intraperitoneal volume, V_D_ (mean values from all patients ± SD, blue dots), and mean simulated profile for the extended model (black solid lines) during 16-h peritoneal dialysis with 7.5% icodextrin.

**Figure 5:**
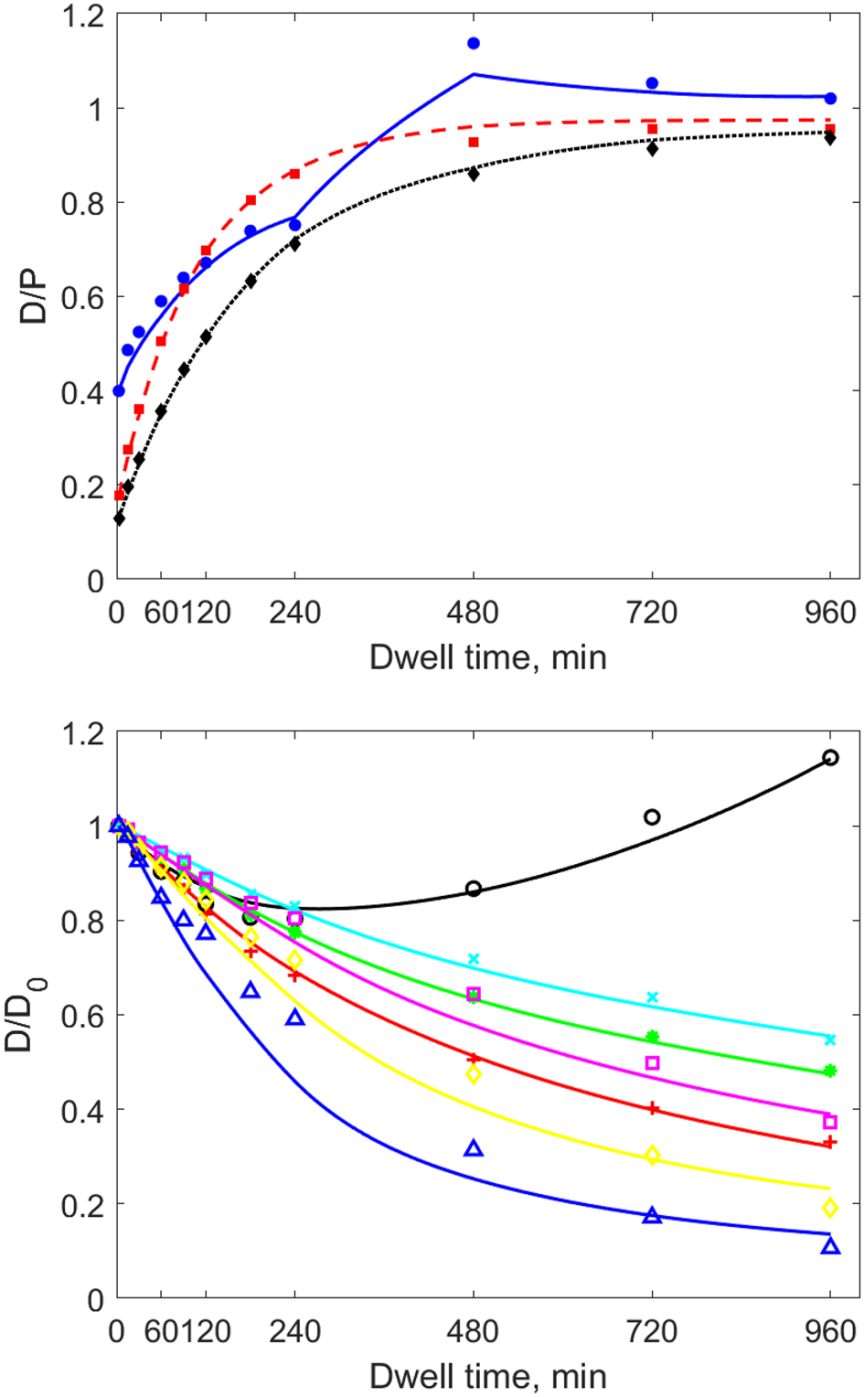
Mean measured and simulated (using the extended model) values from all patients of (upper panel) dialysate to plasma concentration ratio, D/P, for small solutes: glucose (blue circles and solid line), urea (red squares and dashed line), and creatinine (black diamonds and dotted line) and (bottom panel) normalized (by initial value) dialysate concentration, D/D_0_, for LMW icodextrin oligomers (Fraction 1 – circles) and HMW polymers (Fraction 2 – plus marker, Fraction 3 – filled circles, Fraction4 – crosses, Fraction 5 – squares, Fraction 6 – diamonds, Fraction 7–triangles), during 16-h peritoneal dialysis with 7.5% icodextrin. Data are denoted by symbols, whereas lines correspond to the mean numerical simulation.

**Figure 6:**
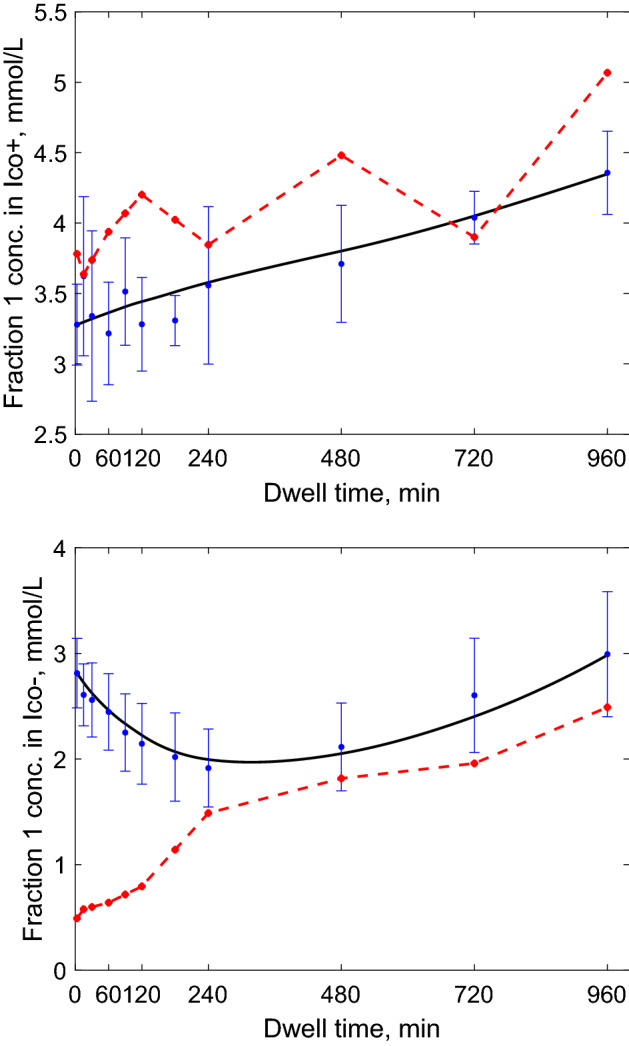
Concentration of LMW icodextrin oligomers (fraction 1) in dialysate during 16-h peritoneal dialysis with 7.5% icodextrin: clinical data for Ico+ (upper panel) and Ico− (bottom panel) group in dialysate expressed as mean ± SD (blue dots) and in plasma as mean (red stars, dashed line) and the mean predicted profile by the extended three-pore model (black solid lines).

The results of numerical simulations showed good agreement with clinical data. The ETPM provides detailed description of the degradation of icodextrin polymers by allowing various hydrolysis patterns of each icodextrin polymer, dependent on icodextrin fractions and amylase concentrations in dialysate. The performance of the model in the prediction of the individual data is presented in Figs. 4 and 5 and in detail in Supplemental Figures 2 and 3 as average data versus average simulated profile calculated over the whole patient group. The mean relative error of fitting for ETPM applied to the individual data was relatively low, 0.062 ± 0.012. The model was able to correctly describe the changes in ultrafiltration volume as shown in Fig. 4 that presents the comparison between data and simulated intraperitoneal volume profile. In case of small solute transport, the dialysate concentration of urea, creatinine and glucose were described in most cases with high precision, including the observed decrease of glucose concentration in dialysate after 480 min related to its decreased concentration in plasma. The corresponding mean data and simulated dialysate to plasma concentration ratios for glucose, urea and creatinine are presented in Fig. 5, upper panel. The differences in the disappearance from the peritoneal cavity among icodextrin fractions are presented in Fig. 5, bottom panel as dialysate concentration over initial concentration for data and numerical results from the extended model. The model, in agreement with clinical data, predicted gradual decrease of concentration of HMW, i.e., icodextrin Fractions 2–7, occurring at different rates. In case of LMW, the model was able to describe properly the different patterns of the changes observed in the concentration of Fraction 1 in dialysate between Ico+ and Ico-  group, Fig. 6. The gradual increase of the concentration of icodextrin polymers from Fraction 1 was observed in patients from Ico+ group during whole dwell period, whereas in Ico− group the decrease of LMW concentration in dialysate was present during initial 2 h followed by the gradual increase after 4 – 6 h of the dwell, Fig. 6.

The transport parameters were similar for Ico+ and Ico− groups and similar to the values typically calculated and applied in TPM. The detailed comparison of mean values obtained for all patients, and Ico+ and Ico− groups separately, and values typically assumed in TPM for glucose-based solutions are presented in Table 1. In addition to the regular TPM parameters the ETPM derives information on the way how dextrins are degraded by α-amylase (described schematically in Fig. 3, see also schematic representation of ETPM concept presented in Supplemental Figure 1). It provides information on the hydrolysis rate constant, $h_{i}$, that describes the α-amylase efficiency to degrade icodextrin Fraction *i* and hydrolysis frequencies/probabilities $p_{i}$ with which polymers from Fraction *i* are selected to be degraded. Interestingly, the obtained values of $h_{i}$ and $p_{i}$ are not equal between fractions, indicating that some forms of degradation processes are more probable than others, as presented in Table 2. There was a tendency for $h_{i}$ rates to be lower for Fractions 4 and 5 in case of All and the Ico− groups, whereas for Ico+ group this tendency was present in Fraction 1 and 4, Table 2. The comparison of the corresponding hydrolysis probabilities in each group showed lower probabilities of hydrolysis for polymers from Fractions 4 and 5 in the All group. However, closer analysis of the obtained values showed that this tendency is present only in Ico− group, whereas in Ico+ group no statistical difference between hydrolysis probabilities was found, Table 2. Analysis of the mean values for Ico+ and Ico− groups suggested faster degradation processes occurring in Ico+ group (in terms of $h_{i}$ rate constants) for LMW polymers (Fraction 1) with a rate that was more than 3 times higher in case of Ico− , whereas at least by half slower degradation processes were found in Ico+ group for Fractions 4–6 compared with Ico− group, Table 2. However, due to the low number of patients in Ico+ group, this finding should be treated only as a possible tendency. The hydrolytic clearances, $H_{i}$, were calculated from the corresponding hydrolysis rates, $h_{i}$ as $H_{i} = V_{D} h_{i} C_{D,amylase}$. Note, that because amylase concentration in dialysate and intraperitoneal volume are changing during the dwell time, $H_{i}$ may change with dwell time, Table 2. The values of $H_{i}$ remained relatively stable during dwell time in Ico+ group, whereas an increase of $H_{i}$ was found in Ico− group, which was statistically significant for all fractions besides Fraction 4 (*p* = 0.13) and Fraction 1 (*p* = 0.052), Table 2. The evaluated hydrolytic clearances were 6–9 times higher (depending on the fraction number) when comparing the initial to final values, Table 2. Moreover, the final values of the hydrolytic clearances estimated for Ico+ group in most cases did not exceed the initial values estimated in Ico− and All groups, or were similar, see Table 2.

**Table 1:** The original (TPM) and extended (ETPM) three-pore model parameters estimated for individual data and expressed as mean ± standard deviation (SD) values calculated for all patients (All, n = 11) and for Ico+ (n = 3) and Ico− (n = 8) groups, where $L_{P} S$ denotes peritoneal ultrafiltration coefficient (hydraulic conductivity), $\alpha_{UP} ,\alpha_{SP} ,\alpha_{LP}$—fractional $L_{P} S$ accounted for by ultrasmall, small and large pores, respectively, L—peritoneal absorption, and $PS$—permeability-surface area product.

Parameter	TPM	Extended TPM model (ETPM)		
		All	Ico+	Ico−
UF coef, L_p_S, mL/min/mmHg	0.075	0.072 ± 0.027	0.053 ± 0.011	0.080 ± 0.027
Pore fractions:				
Ultrasmall, α_UP_	0.02	0.02 ± 0.00	0.02 ± 0.00	0.02 ± 0.00
Small, α_SP_	0.90	0.87 ± 0.02	0.88 ± 0.01	0.87 ± 0.03
Large, α_LP_	0.08	0.11 ± 0.02	0.10 ± 0.01	0.11 ± 0.03
Perit. absorption^1^, L, mL/min	0.30	0.67 ± 0.39	0.79 ± 0.43	0.63 ± 0.40
PS glucose, mL/min	15.40^2^	9.50 ± 4.14	12.30 ± 4.32	8.45 ± 3.53
PS sodium, mL/min	6.00^2^	6.05 ± 0.00	6.05 ± 0.00	6.05 ± 0.00
PS urea, mL/min	14.43^3^	17.87 ± 5.03	15.50 ± 2.67	18.76 ± 5.41
PS creatinine, mL/min	11.99^3^	9.99 ± 3.38	10.70 ± 3.82	9.73 ± 3.16
PS_Ico1_, mL/min	3.96^3^	7.40 ± 2.90	4.07 ± 2.99	8.66 ± 1.56
PS_Ico2_, mL/min	1.56^3^	3.16 ± 1.68	2.47 ± 1.04	3.42 ± 1.80
PS_Ico3_, mL/min	0.58^3^	1.65 ± 1.40	1.35 ± 0.60	1.76 ± 1.59
PS_Ico4_, mL/min	0.19^3^	0.21 ± 0.13	0.30 ± 0.16	0.18 ± 0.09
PS_Ico5_, mL/min	0.04^3^	0.22 ± 0.00	0.39 ± 0.37	0.16 ± 0.13
PS_Ico6_, mL/min	0.005^3^	0.005 ± 0.002	0.008 ± 0.002	0.004 ± 0.001
PS_Ico7_, mL/min	0.002^3^	0.002 ± 0.001	0.002 ± 0.001	0.001 ± 0.001

^1^Peritoneal absorption calculated using volume marker absorption from the peritoneal cavity.

^2^PS assumed in TPM and not derived from the TPM theory^12^.

^3^PS predicted by TPM theory.

**Table 2:** Mean ± SD values of hydrolysis rates, $h_{i}$, hydrolysis probability, $p_{i}$, and time dependent hydrolytic clearances ($H_{i}$ initial and final), related to the degradation caused by α-amylase activity, estimated for the extended three-pore model for all patients (All, n = 11) and for Ico+ (n = 3) and Ico− (n = 8) groups.

Parameter	All	Ico+	Ico−
h_1_, (1/min)/(U/mL)	0.30 ± 0.29	0.12 ± 0.14	0.37 ± 0.30
h_2_, (1/min)/(U/mL)	0.42 ± 0.50	0.33 ± 0.28	0.45 ± 0.56
h_3_, (1/min)/(U/mL)	0.29 ± 0.28	0.23 ± 0.22	0.32 ± 0.30
h_4_, (1/min)/(U/mL)	0.08 ± 0.15	0.12 ± 0.15	0.07 ± 0.15
h_5_, (1/min)/(U/mL)	0.09 ± 0.10	0.21 ± 0.12	0.05 ± 0.03
h_6_, (1/min)/(U/mL)	0.32 ± 0.27	0.50 ± 0.17	0.26 ± 0.27
h_7_, (1/min)/(U/mL)	0.23 ± 0.15^#^	0.23 ± 0.11	0.22 ± 0.16^#^
p_1_	0.23 ± 0.17*	0.08 ± 0.10	0.29 ± 0.16**
p_2_	0.20 ± 0.16*	0.19 ± 0.12	0.21 ± 0.17
p_3_	0.13 ± 0.09*	0.11 ± 0.07	0.14 ± 0.09
p_4_	0.03 ± 0.04	0.04 ± 0.05	0.03 ± 0.04
p_5_	0.06 ± 0.04	0.12 ± 0.02	0.03 ± 0.02
p_6_	0.20 ± 0.12*,**	0.32 ± 0.09	0.15 ± 0.10
p_7_	0.15 ± 0.06*,**	0.14 ± 0.01	0.15 ± 0.07*,**
H_1_, mL/min			
Initial	0.34 ± 0.23	0.15 ± 0.16	0.41 ± 0.21††
Final	2.91 ± 3.81	0.29 ± 0.33	3.89 ± 4.05
H_2_, mL/min			
Initial	0.75 ± 0.85	0.64 ± 0.54	0.79 ± 0.94†
Final	3.66 ± 4.56	1.05 ± 1.00	4.64 ± 4.98
H_3_, mL/min			
Initial	0.42 ± 0.45	0.42 ± 0.42	0.42 ± 0.46†
Final	2.15 ± 2.27	0.72 ± 0.77	2.69 ± 2.42
H_4_, mL/min			
Initial	0.11 ± 0.21	0.22 ± 0.29	0.08 ± 0.15
Final	0.32 ± 0.50	0.39 ± 0.52	0.29 ± 0.49
H_5_, mL/min			
Initial	0.14 ± 0.18	0.36 ± 0.24	0.06 ± 0.04†
Final	0.47 ± 0.34	0.63 ± 0.44	0.41 ± 0.27
H_6_, mL/min			
Initial	0.48 ± 0.45	0.83 ± 0.30	0.35 ± 0.42†
Final	2.41 ± 2.44	1.43 ± 0.64	2.78 ± 2.74
H_7_, mL/min			
Initial	0.35 ± 0.31	0.40 ± 0.22	0.33 ± 0.33†
Final	1.70 ± 1.65	0.69 ± 0.43	2.08 ± 1.77

^#^*p* < 0.05 for post-hoc Tukey test versus h_4_.

*, **p < 0.05 for post-hoc Tukey test versus p_4_ and p_5_, respectively.

^†^*p* < 0.05 and ††*p* = 0.052 for ANOVA repeated measurements applied for the comparison between initial and final value of H_i_ for Ico− group.

In Table 3 mean values of appearance rate constant, $r_{i}^{j}$, are presented for All, Ico− and Ico+ groups describing rates at which hydrolysis of polymer from Fraction *j* leads to the increase (decrease in case of negative values) of molar mass in Fraction *i*. The obtained values showed in general similar values of $r_{i}^{j}$ for $i < j$ and given $j$ that differ from the rate constant corresponding to Fraction $j$, $r_{j}^{j}$. Calculated, negative values of $r_{j}^{j}$, typically observed for higher Fractions 5–7 for All and Ico− groups and Fraction 7 for Ico+ group, suggest domination of hydrolysis of type 1 over type 3 in the high molecular weight Fractions, Table 3.

**Table 3:** Mean ± SD values of appearance rate constants $r_{i}^{j}$ (in (1/min)/(U/mL) describing rate constants at which hydrolysis of polymer *from jth* fraction leads *to* the increase of molar mass in fraction *ith* estimated for all patients (All, n = 11) and for Ico+ (n = 3) and Ico− (n = 8) groups.

From	1	2	3	4	5	6	7
To	All patients, in (1/min)/(U/mL)						
1	0.30 ± 0.29	0.26 ± 0.27	0.08 ± 0.09	0.02 ± 0.02	0.09 ± 0.08	0.27 ± 0.17*	0.43 ± 0.23*
2		0.16 ± 0.36	0.04 ± 0.04	0.02 ± 0.02	0.09 ± 0.08	0.28 ± 0.15*	0.43 ± 0.23*
3			0.17 ± 0.24	0.02 ± 0.02	0.09 ± 0.08	0.28 ± 0.14*	0.43 ± 0.23*
4				0.03 ± 0.17	0.11 ± 0.10^#^	0.29 ± 0.18*	0.43 ± 0.23*
5					− 0.29 ± 0.36	0.35 ± 0.32*	0.43 ± 0.24*
6						− 1.13 ± 0.93	0.51 ± 0.28*
7							− 2.43 ± 1.42
	I+ group, in (1/min)/(U/mL)						
1	0.12 ± 0.14	0.10 ± 0.12	0.03 ± 0.04	0.003 ± 0.002	0.01 ± 0.01	0.07 ± 0.03	0.11 ± 0.06
2		0.23 ± 0.39	0.009 ± 0.005	0.005 ± 0.006	0.03 ± 0.01	0.11 ± 0.03	0.11 ± 0.06
3			0.19 ± 0.24	0.006 ± 0.005	0.03 ± 0.01	0.11 ± 0.03	0.11 ± 0.06
4				0.10 ± 0.14	0.03 ± 0.01	0.11 ± 0.02	0.11 ± 0.06
5					0.12 ± 0.14	0.13 ± 0.04	0.11 ± 0.06
6						− 0.04 ± 0.14	0.12 ± 0.06
7							− 0.44 ± 0.38
	Ico− group, in (1/min)/(U/mL)						
1	0.37 ± 0.30	0.32 ± 0.29	0.10 ± 0.10	0.02 ± 0.02	0.11 ± 0.08*	0.35 ± 0.14*	0.55 ± 0.15*
2		0.13 ± 0.34	0.05 ± 0.04	0.02 ± 0.02	0.11 ± 0.08*	0.34 ± 0.12*	0.55 ± 0.15*
3			0.17 ± 0.24	0.02 ± 0.02	0.11 ± 0.08*	0.34 ± 0.12*	0.55 ± 0.15*
4				0.01 ± 0.17	0.15 ± 0.10*	0.35 ± 0.17*	0.55 ± 0.15*
5					− 0.44 ± 0.29	0.42 ± 0.35*	0.55 ± 0.15*
6						− 1.54 ± 0.75	0.65 ± 0.16*
7							− 1.48 ± 0.42

*p < 0.05 and ^#^0.05 < *p* < 0.1 for difference versus $r_{i}^{i}$ according to post-hoc Tukey test.

## Discussion

There has been a lack of a comprehensive mathematical analysis of peritoneal fluid and solute transport that takes into account the impact of α-amylase on the kinetics of icodextrin fractions and validates kinetic changes of intraperitoneal volumes and solutes, including icodextrin fractions, in patients using icodextrin-based fluid. In the present study, we applied mathematical modelling – combined with detailed clinical and laboratory data—of changes in dialysate volume, solutes (including icodextrin fractions) and α-amylase in 11 patients during 16-h peritoneal dwells with icodextrin-based solutions. The results showed that the model provides accurate (mean relative error of fitting less than 7%) description of peritoneal transport kinetics including that of icodextrin fractions in peritoneal fluid.

Both plasma and dialysate α-amylase underwent major changes. Among the icodextrin-naïve patients, i.e., the Ico− group, the concentration of plasma α-amylase declined throughout the dwell while it remained relatively stable during the whole dwell in the Ico+ group, and at the end of the 16-h dwell the differences between Ico− and Ico+ groups (high at the beginning of the dwell) had almost disappeared, Fig. 2. In contrast, the amylase concentration in dialysate increased 6 times with dwell time in Ico− group and 2 times in Ico+ group, Fig. 3. This pattern of changes indicates that the presence of icodextrin solution in the peritoneal cavity leads to marked changes of α-amylase concentrations in plasma and dialysate. These changes, especially observed in the Ico− group, likely were mediated by corresponding changes in the sub-peritoneal tissues.

The observed changes in the dialysate concentration of icodextrin fractions are the result of the combined impact of dilution, absorption, and α-amylase mediated degradation. An interesting finding in the Ico− group is the novel observation of a U-shaped curve for the dialysate concentration of Fraction 1 over dwell time in the Ico− group (Figs. 4 and 5), but not in the Ico+ group (Fig. 6). The initial decrease is due to the domination of diffusion of oligomers from dialysate into blood and peritoneal tissue and dilution due to ultrafiltration and inflow of icodextrin-free water into peritoneal cavity during the initial dwell period, Fig. 6, bottom panel. The subsequent increase of this icodextrin fraction is the consequence of degradation of larger icodextrin polymers resulting in generation of oligomers. The slow domination of this process over diffusion and dilution, results in the observed increase of concentration within Fraction 1. Finally, the increasing concentration of α-amylase in the dialysate contributes to further increase the concentration of oligomers (Fraction 1) in the dialysate with dwell time. In Ico+ group, the dialysate concentration of oligomers (Fraction 1) increases already from the beginning (Fig. 6) probably mainly due to the initial domination of degradation processes over diffusion from the peritoneal cavity. This is related to the presence of icodextrin oligomers in peritoneal tissue already before the peritoneal exchange, thereby decreasing the effective difference in oligomers concentration (between dialysate and peritoneal tissue and blood), which is a driving force for their diffusion. The presence of oligomers (especially maltose and maltotriose) in the peritoneal tissue and blood in Ico+ group induces diffusion of polymers from Fraction 1 from blood and tissue to the dialysate, whereas in case of their absence in Ico− group diffusion in the opposite direction can be observed, Fig. 6. To better understand the observed phenomenon, a more detailed in-depth analysis of degradation kinetics of each type of dextrins especially in Fraction 1, i.e., dextrins with 2 to 7 D-glucose units, is needed.

To model the changes of icodextrin concentration, the degradation kinetics caused by.α-amylase activity has to be implemented into the mass balance equations. Various approaches have been proposed so far to model enzymatic hydrolysis of starch – from stochastic to deterministic models (using zero-order, first-order kinetics, pseudo-first order kinetics, Michaelis–Menten kinetics etc.^14,29–32^). In contrast to in vitro experiments where the experimental environment is typically well defined and controlled, the icodextrin dynamics analysed in clinical studies might be influenced by additional factors such as changes in amylase concentration, presence of other solutes in the dialysis fluid that may influence amylase activity, and only the net result of peritoneal transport and degradation can be observed and measured. Therefore, in our approach, instead of precise description of the degradation process (patterns), we are focused on the impact of α-amylase activity on the dextrin concentrations, which is similar to the approach proposed by Akonur et al. in which the pseudo-first order kinetics was applied^14^. In the study by Akonur et al., the analysis of degradation pattern was based on in vitro measurements by Nishimura et al. and assumed amylase concentration of 20 U/L that was much higher than the plasma and intraperitoneal concentrations of amylase in our patients, Fig. 2^14,28^. Nishimura et al. observed in vitro the changes of icodextrin distribution (increase of fractions with mass up to 18 kDa and decrease of larger fractions) measured after 5 h and 10 h for various amylase concentrations. Based on these observations, Akonur et al. assumed a decrease of concentration for fractions with mass larger than 10 kDa resulting in the increase of concentration within fractions smaller than 3 kDa (in our case Fractions 1 and 2) based on the ratio 1:3 and with no degradation allowed within the lowest Fractions 1 and 2^14^. As a result, they predicted increase of dialysate concentration of icodextrin Fractions 1 and 2 and minimal decrease within Fraction 3 related to α-amylase activity. Unfortunately, the authors did not provide a quantitative comparison between their predictions and the clinical or experimental data. Moreover, their approach was based on the observation of net changes in icodextrin fraction concentrations, not allowing them to distinguish between different patterns of degradation, in contrast to more recent findings on α-amylase activity patterns^27^. On the other hand, experimental studies in rats showed that the concentration of icodextrin initially increases (up to 60–90 min in Ico+ and Ico− rats) and later decreases within Fraction 1, i.e., MW < 1 kDa, whereas a decrease of the concentration of remaining fractions’ concentrations was observed during the whole dwell period^4^. The discrepancies in the kinetics of Fraction 1 observed between our clinical data and the rat data might be related to the interspecies differences in the level of amylase that is much higher (60 U/L) in rats but also to the α-amylase activity that tends to be lower in CKD patients, especially in case of patients exposed to icodextrin solution^5^. Taking this into account as well as the fact that so far different patterns of icodextrin degradation have been experimentally observed during peritoneal dialysis and proposed to be modelled, we decided to apply the most general approach assuming all possible degradation patterns to estimate corresponding degradation probabilities/frequencies using ETPM of icodextrin kinetics during peritoneal dwell.

Rippe and Levin introduced a new, arbitrary correction, increasing additionally dextrin clearances out of the peritoneal cavity by 1.2 mL/min, to account for their clearance to the peritoneal tissue^12^. However, this assumption has not been qualitatively validated. Due to the lack of data on the accumulation of dextrins in the peritoneal tissue, we decided to not use the correction proposed by Rippe and Levin.

The obtained hydrolysis rates for the extended model ($h_{5} ,h_{6}$ and $h_{7}$) were in All group and Ico− group similar to the degradation rates assumed by Akonur et al. based on in vitro experiments, i.e. 0.08, 0.16 and 0.16 (1/min)/(U/mL), respectively, for Fractions 5, 6 and 7^14^. However, the degradation rate of 0.005 (1/min)/(U/mL) assumed by the authors, which corresponds to our hydrolysis rates in Fractions 3 ($h_{3}$) and 4 ($h_{4}$) together, is by two orders of magnitude lower than the value estimated by us, Table 2. Moreover, whereas Akonur et al. assumed no degradation rate present in Fractions 1 and 2, the estimated degradation rates $h_{1}$ and $h_{2}$ for the extended model were positive in both cases (on average 0.3 and 0.4 respectively in All group, (1/min)/(U/mL)). The observed discrepancies are partially related to the fact that in the study by Akonur et al. an additional clearance out of the peritoneal cavity (1.2 mL/min for humans and 18.2 µL/min for rats) related to the tissue accumulation of icodextrin was assumed as proposed earlier by Rippe and Levin^12,14^,and that the authors were comparing qualitatively their results with rat experiments performed in icodextrin-exposed group, not the icodextrin-naive group^4^. But first of all, the observed discrepancies are related to the differences in the definition between degradation and hydrolysis rates. In our study we evaluated the hydrolysis rates, $h_{{_{i} }}$, that describe the rate at which polymers from Fraction *i* are hydrolysed by α-amylase. This is different from degradation rate, $k_{i}$ in^14^, describing the rate at which polymers from Fraction *i* are hydrolysed to shorter polymers that *belongs to the lower* fractions. Finally, the results of our estimation of hydrolysis rates in Ico+ group suggest tendency for rather higher rates in case of Fractions from 4 to 6, and lower for Fractions 1 and 2, if compared to Ico− group, Table 2.

In our study, for the first time, the accuracy of the three-pore model approach was validated not only with respect to peritoneal transport of water and small solutes but also regarding concentration of icodextrin fractions, reaching a high level of precision with mean relative error being below 0.07. The estimated values of transport parameters were in general similar to the values assumed in TPM as well as to those reported by others, Table 1. In particular, similar values of ultrafiltration coefficient, LpS, estimated in our Ico+ group were found by Galach et al. (0.048 mL/min/mmHg) and by Vonesh et al. using PD Adequest v. 2.0 (0.045 mL/min/mmHg)^2,16^. In the simulations described in the present study, a higher value of peritoneal absorption rate, based on the marker elimination rate from the peritoneal cavity, was used instead of that often assumed for TPM^10,12,17,25,33–35^. In the study by Rippe and Levin, followed by Akonur et al. and Morelle et al., a value of 0.3 mL/min was used, that is much lower than the value, typically around 1 mL/min, as measured using a volume marker^12,14,17^. In the current study, the measured values of peritoneal absorption based on the RISA elimination rate from the peritoneal cavity were on average 0.67, 0.79 and 0.63 for All, Ico+ and Ico− groups, see Table 1. The reported values of peritoneal absorption based on the volume marker, but also other methods of evaluation, remains typically in the range 0.68 – 1.8 mL/min depending on the type of dialysis solution and patient subpopulation^35–40^. Similar values, 0.64 mL/min and 1.03 ± 0.90 mL/min, were applied in previous modelling studies on icodextrin^2,16^. The PS values estimated by us for urea and creatinine were similar to values assumed in TPM and close to values observed by Galach et al. (20.70 mL/min for urea) and Vonesh et al. (19.76 ± 6.96 and 12.11 ± 6.61 mL/min for urea and creatinine, respectively)^2,16^. In the extended three-pore model, the estimated values of PS for glucose were lower than those sometimes assumed for TPM (15.4 mL/min) and closer to the value predicted by the theory of the three-pore model (9.0 mL/min,^25^), and similar to the values estimated from clinical data by Galach et al. (10.53 mL/min) and Vonesh et al. (9.59 ± 5.23)^2,16^. Finally, the estimated values of PS for icodextrin fractions were mostly similar, with respect to interpatient variability, to the values predicted by TPM, except for Fraction 5 for which we got higher values of PS, Table 1. Almost twice higher PS values of icodextrin fractions than those expected from TPM equations were found by Galach et al., being in the range from 4.05 to 0.006 mL/min^16^. Similar values were estimated by Vonesh et al. (from 2.94 ± 1.28 for Fraction 1 to 0.005 ± 0.002 for polymers with MW > 56 kDa)^2^. However, in both mentioned studies, the authors followed the approach by Rippe and Levin and performed all simulations assuming additional clearance of icodextrin fractions equal to 1.2 mL/min (correction to account for absorption to sub-peritoneal tissues) only, thus influencing the estimation of icodextrin PS and degradation rates^2,12,14,16^.

In ETPM, the description of the transport across the peritoneal membrane and peritoneal kinetics of fluid, small solutes and icodextrin fractions was applied according to TPM. Separately, the peritoneal absorption from the cavity was taken into account. The term peritoneal absorption which denotes total absorption from the peritoneal cavity includes (1) absorption from the peritoneal cavity into the adjacent tissue and further by tissue lymphatics to the blood circulation and (2) absorption from the peritoneal cavity directly into lymphatics (mainly by diaphragmatic lymphatics open to peritoneal cavity). The direct absorption was estimated to account for only 10–30% of total absorption and is therefore insufficient to describe correctly fluid and solute peritoneal kinetics^41,42^. In ETPM, following the approach by Flessner and others, the rate of total peritoneal absorption of fluid and solutes from the peritoneal cavity was assumed based on the measurements of a volume marker^16,33,35,38,40,41^. Such an approach gives a correct description of the absorption of the volume marker and guarantees that the obtained/simulated absorption of larger molecules (for example icodextrin) is correct^16,33,38^. Moreover, the usage of direct peritoneal absorption (for example lymphatic flow L = 0.3 mL/min proposed by Rippe et al.) instead of the total peritoneal absorption requires additional ad hoc inflation of PS for glucose and icodextrin clearances to increase their clearances from the peritoneal cavity over PS values calculated from the TPM theory^12,25^.

It should be pointed out that both the original and extended three-pore approaches allow for the calculation of the aggregated peritoneal water and solute transport between blood circulation and peritoneal cavity. According to these approaches the peritoneal barrier is treated as a semipermeable membrane not distinguishing between capillary wall and tissue barrier. To look separately at the transport through the peritoneal tissue, a more complex approach has to be applied such as the distributed model^9,43,44^. During peritoneal dwells with icodextrin-based solution dextrins accumulate in the tissue, but they are also degraded there by α-amylase present in the interstitial fluid of peritoneal tissue at higher levels than in the peritoneal cavity. Moreover, one might expect higher rates of degradation and accumulation in Ico− group than in Ico+ group due to the initial absence of dextrins and higher levels of amylase in plasma and the peritoneal tissue in the Ico− group. Due to the lack of data on the processes of icodextrin accumulation and degradation in the peritoneal tissue, and to avoid impact of the mentioned correction on the estimated parameters, we decided to use the original TPM approach in both Ico+ and Ico− groups and to estimate PS values of icodextrin fractions that represent their disappearance from the peritoneal cavity by diffusion.

The impact of α-amylase on the peritoneal kinetics of icodextrin is well reflected in the estimated values of hydrolytic clearances, $H_{i}$, as calculated by ETPM. The comparison of $H_{i}$ with $PS_{{Ico_{i} }}$ for icodextrin fractions (reflecting the maximal rates of their clearance by diffusion) showed that in case of shorter polymers (from Fractions 1, 3 and 2 in most cases) diffusive clearance dominates over degradation processes during the whole dwell time in Ico+ as well as in Ico− groups, Tables 1 and 2. In case of Fractions 4 and 5, the initial domination of diffusive clearance slowly decreases during the dwell time, being dominated by hydrolytic clearance at the end of the exchange, at least partly due to the changes in α-amylase concentration and intraperitoneal volume, see Tables 1 and 2. In case of HMW polysaccharides from Fractions 6 and 7, their hydrolytic clearances remain higher than the diffusive ones during whole dwell time. Interestingly, the obtained values of hydrolytic clearances in Ico+ group typically remain below 1.2 mL/min except for Fraction 6, for which $H_{6}$ increased from initial value of 0.83 ± 0.30 to 1.43 ± 0.64 mL/min at the end of the peritoneal dwell, Table 2. On the other hand, the estimated final values of hydrolytic clearances in Ico− group were on average higher than 1.2 mL/min in all fractions except Fractions 4 and 5, Table 2. Moreover, estimated frequencies of icodextrin fractions hydrolysis, $p_{i}$, suggested that the degradation of dextrin with MW in the range from 21.4 kDa to 43.5 kDa (Fraction 5) is less likely, especially in case of Ico− group, Table 2. Whereas the low number of patients in Ico+ group does not allow a proper statistical comparison between the two Ico groups, the mean values of parameters in Table 2 showed a tendency for slower degradation rates in Ico+ group for lower icodextrin fractions 1–3, whereas faster degradation rates were found for polymers from Fractions 4–6, Table 2. These findings regarding differences in amylase activity are in line with previous data showing lower efficiency of α-amylase in patients with end-stage kidney disease and even lower in PD patients treated with icodextrin^5^. Moreover, our data showed that in the Ico+ group the concentration of α-amylase in plasma and dialysate as well as hydrolytic clearances were more stable with less changes during the dwell time, Fig. 2 and Table 2. In addition, results from the extended model showed that degradation patterns differed between the two Ico groups, Table 2. This finding might be also related to the different concentration profiles in Ico+ and Ico− groups that were measured for icodextrin Fraction 1, Fig. 6. However, irrespectively of the differences in the concentration of α-amylase and icodextrin fractions between Ico+ and Ico− group, the corresponding ultrafiltration (UF) values remained similar^18^. Different factors besides amylase activity, also influence overall water removal. Transport properties of the peritoneal membrane such as ultrafiltration coefficient, osmotic conductance, peritoneal absorption and PS values, which were numerically different between the two studied groups, contribute to the overall UF as well. There was a tendency for lower ultrafiltration coefficient, lower osmotic conductance and higher peritoneal absorption leading to lower UF in the Ico+ group compared with Ico− group, although PS values for lower icodextrin fractions (longer persistence of the osmotic gradient) were lower in Ico+ group. The relatively large variability within each group as well as the complexity of the network of transport and hydrolysis factors, parameters and processes contributing to water removal makes it difficult to explain, based on our data, the observed UF rate in Ico+ vs. Ico− groups (especially considering that these groups included different patients). Further studies are needed in this area.

In the extended model, detailed kinetics of icodextrin degradation by amylase was taken into account. We did not assume any specific pattern of degradation considering that amylase may hydrolyse polymers belonging to each fraction and that the probability of getting polymers belonging to the same or lower fraction can be positive. This approach seems to be more reasonable and in agreement with experimental data on different patterns of amylase activity^27^. This allowed us to describe degradation rates resulting from the degradation of polymers from various icodextrin fractions, and therefore dependent on the concentration of these fractions.

The extended three-pore model (ETPM), which incorporates the hydrolysis of icodextrin fractions and thereby provides the ability to quantify related processes, increases our knowledge on the implications of icodextrin degradation and our understanding of the processes leading to the observed changes within the osmotic gradient during the peritoneal dwell. The model can also be applied for further investigations of possible differences in icodextrin hydrolysis between icodextrin-naive and icodextrin-exposed patients.

One limitation of the present study is the low number of investigated patients and the unbalanced distribution of patients in the Ico+ (n = 3) and Ico− (n = 8) groups. This was because data on changes within each fraction’s concentration in dialysate and plasma were available in only 11 patients and, at the time of the study, there were only three long term icodextrin-exposed patients in the clinical centre where the study was carried out. Therefore, further studies are needed to provide more insights into the possible differences between the two groups.

Meanwhile, as the extended model properly describes peritoneal kinetics in both Ico+ and Ico− groups, it can be applied to predict removal of solutes and excess water, and treatment efficiency, in icodextrin-naive as well as in icodextrin-exposed patients. Furthermore, our model can be used to simulate different daily PD schedules – including incremental dialysis—with icodextrin and glucose-based solutions to select schedules that optimize patient’s treatment in terms of solute and fluid removal. Further studies are warranted for example to investigate the impact of replacing glucose dwells by icodextrin dwells and verification of the model´s predictions.

## Conclusions

Modelling of icodextrin kinetics during peritoneal dialysis—based on detailed clinical and laboratory data including α-amylase activity in dialysate—provided accurate description of peritoneal transport of fluid and solutes (includinf icodextrin fractions) during long icodextrin dwells. In addition, quantitative analyses of factors—dilution, convective absorption and diffusion out of the peritoneal cavity, as well as α-amylase mediated degradation – simultaneously contributing to the changes of icodextrin fractions concentration and osmotic driving forces provided the patterns of icodextrin degradation in the peritoneal cavity.

The applied model provided new insights regarding mechanisms that may explain the observed differences in degradation dynamics between icodextrin-naïve and icodextrin-exposed patients as well as novel information on the pattern of icodextrin degradation by α-amylase. This information may be helpful in clinical settings when analysing data on the impact of changes in the composition of icodextrin fractions on kinetics of peritoneal transport in patients using icodextrin-based solutions.

## Supplementary Information

Supplementary Information.

## Data Availability

A detailed description of the mathematical model used in this study and figures showing more details on the goodness of the model to describe clinical data may be found in the Supplementary material.
