# Supplementary material for: Modelling of icodextrin hydrolysis and kinetics during peritoneal dialysis
Source: Sci Rep. 2023 Apr 21;13:6526. doi: 10.1038/s41598-023-33480-w (PMC10121670; doi:10.1038/s41598-023-33480-w)
Supplement: Supplementary file 1 — Supplementary Information. [file 41598_2023_33480_MOESM1_ESM.doc]

**Supplementary material**

**Modelling of icodextrin hydrolysis and kinetics during peritoneal dialysis**

Joanna Stachowska-Pietka, Jacek Waniewski, Anna Olszowska, Elvia Garcia-Lopez, Zofia Wankowicz, Bengt Lindholm

*Estimation of icodextrin metabolites distribution*

The molecular weight and distribution of icodextrin polymers according to MW were estimated assuming log-normal distribution of MW, as proposed previously in ^1^, and further applied to calculate cumulative number-average (Mn) and weight-average (Mw) molecular weights for each icodextrin fraction as well as the corresponding effective radii using empirical formula of Mark-Houwink-Kuhn-Sakurada $r_{s}\text{ }=\text{ }0.486MW^{0.385}$ ^2^. Rippe et al. showed that increase of the number of representative fractions above seven does not improve prediction of the ultrafiltration ^2,3^. Therefore, we decided to use seven reference fractions (instead of using whole MW distribution) as shown in **Figure 1**. Using the procedure described above we obtained a number-average molecular weight, Mn, and the weight-average molecular weight, Mw, for fresh icodextrin solution of 5 kDa and 11 kDa, respectively with polydispersity index DP=2.2 and net molarity of 15.2 mmol/L (12 mmol/L as reported in previous studies ^1,4^). The accuracy of data fitting to the log-normal distribution was high with R^2^=0.998.

*Three-pore model for icodextrin peritoneal transport*

The schematic concept of the extended three-pore model (ETPM) is presented in **Supplemental Figure 1**. In the ETPM, the description of peritoneal kinetics of fluid, small solutes and icodextrin fractions was taken according to the original three-pore model (TPM) with the transport across the peritoneal membrane and, separately, the peritoneal absorption from the cavity taken into account and to this was added the impact of the hydrolysis of icodextrin. The TPM for peritoneal transport was applied as proposed by Rippe et al. ^2^. Briefly, the change of intraperitoneal volume ($V_{D}$, mL) per unit time was described using the following equation:

$$\frac{dV_{D}}{dt}=J_{V,UP}+J_{V,SP}+J_{V,LP}-L$$

where $J_{V,UP},\text{ }J_{V,SP},\text{ and }J_{V,LP}$ (mL/min) were water flows through the transcellular, small, and large pores into the peritoneal cavity, respectively and $L$ (mL/min) stands for peritoneal (lymphatic) absorption. The changes of small solute concentrations in dialysate ($C_{D,S}$, mmol/L) can be calculated from the mass balance equation as follows:

$$\frac{d\left(C_{D,S}\right)}{dt}=\frac{1}{V_{D}}\left(J_{S,SP}+J_{S,LP}-C_{D,S}\left(J_{V,UP}+J_{V,SP}+J_{V,LP}\right)\right)$$

where $J_{S,SP}\text{ and }J_{S,LP}$ (mmol/min) corresponds to the solute flow through small and large pores into the peritoneal cavity, respectively, and index $S$ stands for glucose, urea, creatinine, and sodium, respectively.

The fluid flow over each pore pathway (UP, SP, or LP) can be calculated from the pore theory as:

$$J_{V,pore}=\alpha_{pore}L_{p}S\left[\Delta P-\left(\Delta O_{S,pore}+\Delta O_{Ico,pore}+\Delta O_{Onc,pore}\right)\right],$$

where $\alpha_{pore}$ describes contribution of each pore to the peritoneal barrier hydraulic conductance, $L_{p}S$. The hydraulic pressure gradient, $\Delta P$ , between peritoneal cavity and blood was assumed to be initially 8 mmHg and later on adapted following changes in the intraperitoneal volume ^2^. The overall osmotic pressure gradient (including oncotic) was calculated as a sum of osmotic pressure exerted by small solutes ($\Delta O_{S}$, calculated for glucose, sodium, urea, and creatinine), oncotic pressure gradient ($\Delta O_{onc}$, exerted by proteins), and colloid pressure gradient exerted by icodextrin metabolites ($\Delta O_{Ico}$, estimated for representative icodextrin fractions), all calculated for each type of pore. The reflection coefficient, $\sigma_{pore}$ , was calculated for each type of the pore and each solute, and the effective oncotic pressure gradient was calculated assuming reflection coefficient for albumin and the oncotic pressure of 22 mmHg ^2^.

In case of small solutes, the flow of solutes through small and large pores was calculated as follows:

$$J_{S,pore}=PS_{S,pore}(C_{B,S}-C_{D,S})+J_{V,pore}(1-\sigma_{S,pore})\bar{C}_{S,pore}=J_{V,pore}(1-\sigma_{S,pore})\frac{C_{B,S}-C_{D,S}e^{-Pe_{S,pore}}}{1-e^{-Pe_{S,pore}}}$$

where $C_{B,S}$ and $\bar{C}_{S,pore}$ are solutes concentration in plasma and mean concentration across the peritoneal membrane, respectively, and parameters $PS_{S,pore}$ and $Pe_{S,pore}$ stands for diffusive permeability of the peritoneal membrane and the Peclet number, respectively both calculated from the pore theory ^2^.

Due to the long duration of the peritoneal dwell, the assumption of the classical 3PM that plasma concentrations of solutes remain constant during dwell time was not fulfilled. Due to variability of small solute concentrations in plasma over dwell time (especially seen in case of glucose and sodium), the 3PM was extended and the individual, time dependent profiles were taken into account in the 3PM simulations.

*Numerical simulations for ETPM*

The goodness of fit of the ETPM are visualized in **Supplemental Figures 2-3**, where mean numerical profiles from the simulations were compared with the mean experimental data.

**Supplemental Figure 1:** A scheme presenting the extended three-pore model concept with the description of fluid and solutes transport following original three-pore model (TPM; solid box) and its extension to include also icodextrin kinetics due to the hydrolysis of icodextrin fractions (dashed box, presented schematically in **Figure 3**).

**Supplemental Figure 2:** Measured values of intraperitoneal volumes and small solute concentrations in dialysate: glucose, urea, creatinine, and icodextrin low molecular weight (Ico1) oligomers (mean values from all patients ± SD, blue dots) and plasma (mean values, red crosses) and simulation profiles for the extended (black solid lines) model during 16-hour peritoneal dialysis with 7.5% icodextrin.

**Supplemental Figure 3:** Measured values of icodextrin HMW metabolites concentrations (Fractions 2-7, mean values from all patients ± SD, blue) and simulation profiles for the extended (black solid lines) model in dialysate during 16-hour peritoneal dialysis with 7.5% icodextrin.

**References**

1 Vonesh, E. F., Story, K. O., Douma, C. E. & Krediet, R. T. Modeling of icodextrin in PD Adequest 2.0. *Perit Dial Int* **26**, 475-481 (2006).

2 Rippe, B. & Levin, L. Computer simulations of ultrafiltration profiles for an icodextrin-based peritoneal fluid in CAPD. *Kidney Int* **57**, 2546-2556, doi:10.1046/j.1523-1755.2000.00114.x (2000).

3 Morelle, J. *et al.* Mechanisms of Crystalloid versus Colloid Osmosis across the Peritoneal Membrane. *J Am Soc Nephrol* **29**, 1875-1886, doi:10.1681/ASN.2017080828 (2018).

4 Leypoldt, J. K., Hoff, C. M., Akonur, A. & Holmes, C. J. Low-Polydispersity Glucose Polymers as Osmotic Agents for Peritoneal Dialysis. *Perit Dial Int* **35**, 428-435, doi:10.3747/pdi.2013.00232 (2015).
